# Ginkgo Biloba and Long COVID: In Vivo and In Vitro Models for the Evaluation of Nanotherapeutic Efficacy

**DOI:** 10.3390/pharmaceutics15051562

**Published:** 2023-05-22

**Authors:** Thelma Akanchise, Angelina Angelova

**Affiliations:** Université Paris-Saclay, CNRS, Institut Galien Paris-Saclay, 91400 Orsay, France; thelma.akanchise@universite-paris-saclay.fr

**Keywords:** Ginkgo biloba bioactive compounds, neuroinvasive coronavirus infection, neurological long COVID, oxidative stress, neuroinflammation, anti-inflammatory and anti-apoptotic agents, nanotherapy, nanoparticle therapeutic efficacy, in vivo and in vitro models

## Abstract

Coronavirus infections are neuroinvasive and can provoke injury to the central nervous system (CNS) and long-term illness consequences. They may be associated with inflammatory processes due to cellular oxidative stress and an imbalanced antioxidant system. The ability of phytochemicals with antioxidant and anti-inflammatory activities, such as Ginkgo biloba, to alleviate neurological complications and brain tissue damage has attracted strong ongoing interest in the neurotherapeutic management of long COVID. Ginkgo biloba leaf extract (EGb) contains several bioactive ingredients, e.g., bilobalide, quercetin, ginkgolides A–C, kaempferol, isorhamnetin, and luteolin. They have various pharmacological and medicinal effects, including memory and cognitive improvement. Ginkgo biloba, through its anti-apoptotic, antioxidant, and anti-inflammatory activities, impacts cognitive function and other illness conditions like those in long COVID. While preclinical research on the antioxidant therapies for neuroprotection has shown promising results, clinical translation remains slow due to several challenges (e.g., low drug bioavailability, limited half-life, instability, restricted delivery to target tissues, and poor antioxidant capacity). This review emphasizes the advantages of nanotherapies using nanoparticle drug delivery approaches to overcome these challenges. Various experimental techniques shed light on the molecular mechanisms underlying the oxidative stress response in the nervous system and help comprehend the pathophysiology of the neurological sequelae of SARS-CoV-2 infection. To develop novel therapeutic agents and drug delivery systems, several methods for mimicking oxidative stress conditions have been used (e.g., lipid peroxidation products, mitochondrial respiratory chain inhibitors, and models of ischemic brain damage). We hypothesize the beneficial effects of EGb in the neurotherapeutic management of long-term COVID-19 symptoms, evaluated using either in vitro cellular or in vivo animal models of oxidative stress.

## 1. Introduction

**Figure 1:**
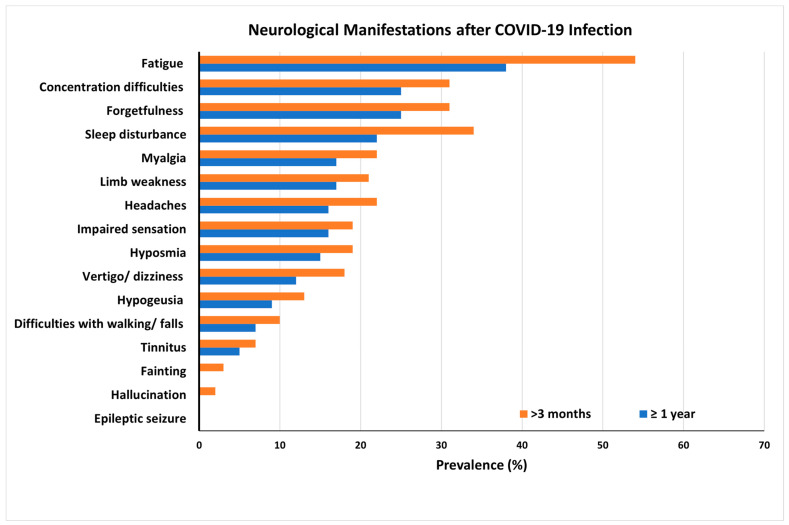
Long COVID symptoms and prevalence of long-term COVID-19 neurological complications in patients at risk following coronavirus infection. Results obtained from a global online survey. The most frequent symptoms persisted after 3 and 12 months in 216 respondents from a cohort of suspected and confirmed COVID-19 cases. Adapted with permission from [11]. Copyright {2022} Science-HHS Public Access (PubMedCentral).

Long COVID involves continuous, long-term manifestations of the residual damages and sequelae of coronavirus infection caused by human severe acute respiratory syndrome coronavirus 2 (SARS-CoV-2), a large positive-stranded enveloped RNA virus that generally provokes respiratory diseases [1,2]. SARS-CoV-2 predominantly affects the respiratory system but can also invade the nervous system and cause multiple neurological disorders [3,4,5]. Patients at risk following COVID-19 infection may still exhibit a range of long-term neurologic and psychiatric disorders (Figure 1) and may not fully recover several months post-infection. These sequelae include mild symptoms, such as headaches; extreme tiredness (fatigue); loss of smell, taste, or tactile sensing functions; cognitive impairment; depression; delirium; and psychosis [5]. More severe documented outcomes include cases of encephalitis, Guillain–Barre syndrome, and stroke [5]. These long-term neurological problems following SARS-CoV-2 infection have been described as manifestations of post-COVID-19 syndrome, long COVID, long haulers, or the post-acute sequelae of SARS-CoV-2 (PASC) [6,7]. A study performed in Italy found that 87.4% (*n* = 179) of patients who recovered from COVID-19 still report the persistence of at least one symptom, particularly fatigue and dyspnea [8]. Another work reported that the most prevalent symptoms of patients in the 6 months following hospitalization were tiredness (34%), memory/attention problems (31%), and sleep disturbances (30%). Neurological abnormalities were found in 40% of patients who underwent a neurological examination, including hyposmia (18.0%), cognitive impairments (17.5%), postural tremor (13.8%), and mild motor/sensory deficits (7.6%) [9]. Moreover, postmortem studies have established brain tissue edema and partial neuronal degeneration in deceased patients [10].

Due to their neuroinvasive nature, coronaviruses may invade the central nervous system (CNS), causing inflammation and demyelination [12]. According to recent studies, SARS-CoV-2 can reach the CNS in many different ways, including (i) the hematopoietic pathway and subsequent blood–brain barrier (BBB) rupture, (ii) blood–cerebrospinal fluid (B-CSF) distribution, (iii) transsynaptic viral spreading from the peripheral nerve, (iv) via circumventricular organs (CVOs), and (v) olfactory bulb penetration due to the interaction between the virus spike 1 (S1) protein and the angiotensin-converting enzyme 2 (ACE2) receptor [3,13,14]. The latter is widely expressed in neurons, oligodendrocytes, and astrocytes throughout the brain. Additional evidence of these entry pathways has been obtained using genome sequencing and via viral detection in the cerebrospinal fluid (CSF) of several patients [15].

Scientific literature points out three factors that favor the pathogenesis of long COVID: neuroinflammation, thrombosis, and immunosuppression [16,17]. For neurological long COVID sequelae, oxidative stress appears to be the major underlying mechanism. Concurrently, inflammation and thrombosis contribute to reactive oxygen species (ROS) reactivation, resulting in a vicious cycle of oxidative stress, inflammation, and disease progression. According to in vitro studies, oxidative stress is a factor in NLRP3-mediated IL-1 release by monocyte cells exposed to SARS-CoV-2 [17]. There is evidence that oxidative stress, caused by an increase in ROS generation after hypoxia, leads to the apoptosis and cellular death of dopamine-containing neurons (DCNs). As a result, Parkinson’s disease (PD) may exacerbate as a severe neurodegenerative disorder [18].

**Figure 2:**
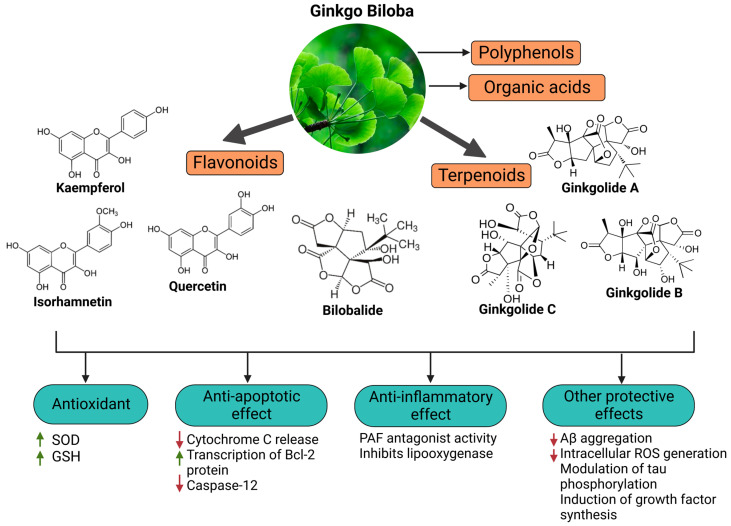
Primary bioactive compounds in Ginkgo biloba and their pharmacological effects (created with *BioRender* using information from [21,22]). Up and down arrows indicate the upregulation and downregulation of relevant biomarkers, respectively.

Since oxidative stress is intertwined with the onset and pathogenesis of the post-acute sequelae of SARS-CoV-2 infection, harnessing this pathway is a step toward finding new therapeutic options in response to this healthcare challenge. Anti-inflammatory and antioxidant agents may efficiently reduce the complications experienced by patients post-COVID-19. In this context, phytochemicals, such as Ginkgo biloba leaf extract (EGb), have demonstrated significant potential as antiviral agents targeting various stages of the coronavirus life cycle [19,20]. The active constituents of EGb include flavonoids (e.g., quercetin, kaempferol, and isorhamnetin), biflavones (sciadopitysin and ginkgetin), terpene trilactones (ginkgolides and bilobalide), and ginkgolic acids (alkylphenols); see Figure 2.

It has been shown that EGb 761, a standardized extract that contains bilobalide, 24% flavone glycosides (quercetin, kaempferol, and isorhamnetin), and 6% terpenes, increases the synthesis of INF-γ, while reducing the release of pro-inflammatory cytokines in peripheral blood leukocytes [22]. Docking simulations and inhibition kinetic studies have demonstrated that ginkgolic acids and bioflavonoids derived from Ginkgo biloba exhibit comparatively potent SARS-CoV-2 3CLpro inhibitory capabilities. Thus, several promising leading compounds have been identified for the advancement of antiviral medication research by targeting the 3CLpro enzyme [19]. However, there is currently limited scientific literature on the possible use of EGb nanotherapy for managing long-term neurological problems related to SARS-CoV-2 or an overview of several models to evaluate the therapeutic outcome of EGb. Recent studies on Ginkgo biloba have examined the efficiency of various EGb extracts against age-related disorders, including dementia, Alzheimer’s disease, and mild cognitive impairment (MCI). These studies include those by Barbalho et al., Singh et al., and Tomino et al. [23,24,25]. Al-Kuraishy et al., however, discussed the use of EGb in managing COVID-19 severity but did not thoroughly review the effectiveness of EGb nanotherapies [26].

This review gives a comprehensive overview of the potential uses of Ginkgo biloba as an antiviral, anti-inflammatory, and antioxidant agent capable of regulating the neurological complications of SARS-CoV-2 infection. We summarize the reported findings of in vivo and in vitro models mimicking oxidative stress conditions and aiming at the discovery of novel nanotherapeutics based on Ginkgo biloba ingredients. We hypothesize that the presented results can be of interest to the neurotherapeutic management of long COVID.

## 2. Long-Term Neurological Damage and the Role of Oxidative Stress

### 2.1. Neuroinvasive and Neurotoxic Potential of Coronavirus Linked to Neurodegeneration

The CNS has been proven to be susceptible to viral infections [3,4,27,28]. A series of recent studies have demonstrated the neuroinvasive potential of SARS-CoV-2, like the previously established neurovirulence of human coronaviruses, such as SARS-CoV, MERS-CoV, HcoV-229E, and HcoV-OC43 [28,29,30,31,32]. According to Boroujeni et al., COVID-19 impacts the cerebral cortex of patients. This effect is characterized by activated microglia that amplify the inflammatory activation of astrocytes and is accompanied by low glutathione levels and upregulation of inflammation [33]. Palpagama et al. asserted that the activation of glial cells is involved in the pathology of neurodegenerative disorders [34]. The elevated number of such cells can be associated with neuroinflammation and brain tissue damage.

Other researchers have detected the presence of SARS-CoV-2 RNA in the cerebrospinal fluid after the virus infects the patient’s CNS and causes meningitis and encephalitis [10,32,35,36]. Alternatively, evidence has revealed that 50% of patients with COVID-19 develop intestinal inflammation [37,38,39]. These results support the gut-driven inflammation hypothesis of Parkinson’s disease (PD) pathogenesis, which starts in the intestines and advances through inflammation toward the CNS. The associated increased levels of α-synuclein initiate aggregation in the gut and brain [40]. Magnetic resonance imaging (MRI) data have also shown brain alteration in the cortical region (posterior gyrus rectus) that is associated with olfaction. This fact suggests that SARS-CoV-2 can invade the brain through the olfactory pathway and cause olfactory dysfunction, among other neurological disorders [41]. Additionally, olfaction-related brain changes in the posterior gyrus rectus of the brain have been observed using magnetic resonance imaging (MRI) [41]. This finding suggests that SARS-CoV-2 can enter the brain via the olfactory pathway and induce neurological diseases, such as olfactory impairment and other neurological conditions.

**Figure 3:**
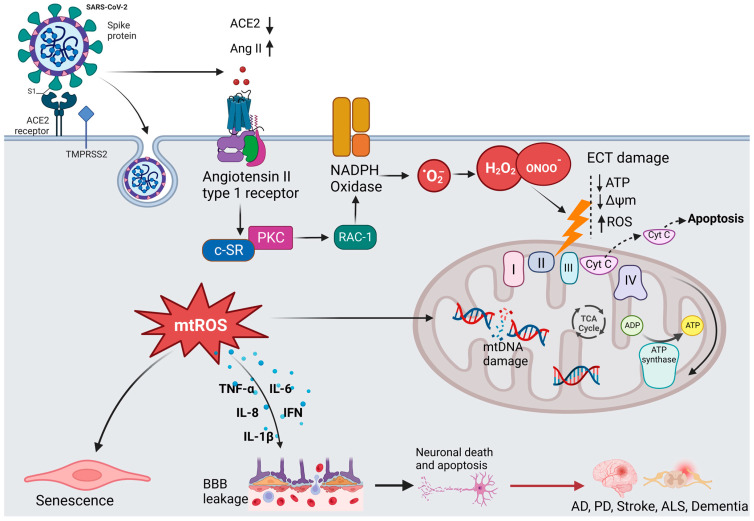
The hypothesized mechanism of coronavirus-induced neuronal damage via oxidative stress and mitochondria dysfunction. SARS-CoV-2 infection occurs when TMPRSS2 primes the spike proteins for proteolysis, allowing the binding to ACE2. The affinity interaction triggers the binding of Ang II to the angiotensin type 1 receptor (AT1R), activating NADPH oxidase. This leads to mitochondrial electron transport chain (ETC) damage via the release of oxidative and nitrosative species, subsequently increasing the formation of mitochondrial reactive oxygen species (mtROS). The signaling pathways mediated by mtROS trigger the production of inflammatory cytokines, which can compromise the blood–brain barrier, thus resulting in neuronal damage. Additionally, mtROS cause nuclear and mitochondrial damage, which prolongs mitochondrial dysfunction and encourages inflammatory senescence (created with BioRender).

It is hypothesized that the neuroinvasive potential of SARS-CoV-2 plays a subordinate role in the pathogenesis of long COVID [4,42,43,44]. The virus enters the CNS and the PNS via the engagement of hematogenous or transsynaptic pathways (through the nasal cavity or the bloodstream) and triggers neuroinflammation [45]. As shown in Figure 3, SARS-CoV-2 invades the host cells by binding to the ACE2 receptor with its spike (S) protein and then priming with the S protein through the activities of the transmembrane protease, serine 2 (TMPRSS2) [46]. ACE2 is highly expressed in the brain of humans and animals, notably in some brain locations, such as the choroid plexus and paraventricular nuclei of the thalamus and in non-neuron cells (mainly astrocytes and oligodendrocytes) as well as various vessel calibers in the frontal cortex [47]. The presence of ACE2 in primary human brain microvascular endothelial cells (hBMVECs) alleviates the ability of SARS-CoV-2 to compromise the activity of the BBB [47,48]. Coronavirus infections are primarily associated with cytokine production, inflammation, and apoptosis in accordance with the pathophysiological process of oxidative stress [49].

### 2.2. Oxidative Stress and Redox Signaling, Players in SARS-CoV-2 Neurological Damage

It has been shown that Parkinson’s disease and SARS-CoV-2 infection cause similar oxidative stress and the activation of nuclear factor kappa B (NF-κB) [18,50,51]. In addition, activating pro-inflammatory mediators, such as IL-1 and IL-6, can lead to amyloid beta (Aβ) deposition and accumulation, thus establishing a link between neuroinflammation and Alzheimer’s disease [14,52,53].

It has been demonstrated that the receptor-binding domain (RBD) of the SARS-CoV-2 spike 1 (S1) protein binds to Aβ and tau proteins and causes their aggregation [54]. Additionally, SARS-CoV-2 infection has been linked to hypoxia and decreased oxygen levels [53]. The mitochondria of brain cells may undergo a rise in anaerobic metabolism due to this process, which can increase the levels of lactic acid, lipid peroxides, and oxygen-free radicals and deplete the antioxidant system. Consequently, the BBB is compromised, which may result in CNS complications. [36,49].

In regard to the role of oxidative stress in chronic diseases, Aranda-Rivera et al. highlighted the importance of nuclear factor erythroid 2–related factor 2 (Nrf2), a ubiquitous protein (containing 605 amino acids) that can modulate cellular oxidative stress [55]. During oxidative stress, Keap1 undergoes conformational change due to the oxidation of residues Cys 226, Cys 613, and Cys 624 by electrophiles and oxidants [56]. This mechanism enables Nrf2 to escape ubiquitination, leading to its release into the nucleus and regulating the expression of a number of antioxidant and detoxifying enzymes, such as glutamate-cysteine ligase modifier (GCLM) subunits, heme oxygenase, NAD(P)H quinone dehydrogenase 1 (NQO1), glutathione S-transferase, and glutathione peroxidase [57,58].

It should also be emphasized that viral infections, such as SARS-CoV-2, have adverse effects on antioxidant systems and have been linked to phenomena involving the inhibition of Nrf2 and activation of the NF-kB pathway in favor of inflammation and oxidative stress. Olagnier et al. showed that during SARS-CoV-2 infection, the Nrf2 pathway is repressed, leading to the downregulation of heme oxygenase 1 (HO-1) and NQO1 [59]. Therefore, several antioxidant enzymes that guard against oxidative stress, including glutathione peroxide, peroxiredoxin, thioredoxin reductase, and thioredoxin, are affected.

Numerous studies have shown that the phosphatidylinositol 3-kinase (PI3K)/AKT signaling pathway controls oxidative stress by activating the transcription factor FOXO3 and initiating the transcription of antioxidant proteins, such as SOD-2, peroxiredoxins (PRDXs) 3 and 5, which are found in the mitochondria, and catalase, which is found in the peroxisomes [60,61,62]. Peroxisome-proliferator-activated receptor coactivator 1 (PGC-1), the master biogenesis regulator that promotes the transcription of antioxidant enzymes, interacts with the FOXO3 transcription factor to control oxidative stress in the mitochondria [61]. However, research has revealed that the ability of PGC-1 to promote gluconeogenesis and fatty acid oxidation is inhibited by protein kinase Akt2/protein kinase B (PKB), which acts as an intermediary trigger of phosphorylation and inhibition [62].

NF-κB is another transcription factor that regulates stress responses. It is activated by the phosphorylation of I-κB, thanks to the I-κB kinase (IKK) complex. In vitro studies by Wu et al. have reported that sustained exposure of human lens epithelial cells (HLECs) to increasing doses of H_2_O_2_ (50–100 μM) for 4 h attenuates the TNFα-induced degradation of I-κB, accompanied by the activation of NF-κB and proteasome activity by 50–80% [63]. The obtained data have also indicated that the activation of NF-κB is an essential phenomenon that enables cells to recover from oxidative stress.

### 2.3. In Vivo and In Vitro Models of Oxidative Stress

Over the years, various oxidative stress models have been developed to study the pathogenesis of neurodegenerative disorders and discover new strategies for developing therapeutic agents. Such experimental techniques include the use of lipid peroxidation products, endogenous antioxidant depletion, mitochondrial respiratory chain inhibitors, neurotoxic agents (e.g., rotenone and *N*-methyl-4-phenyl-1,2,3,6-tetrahydropyridine (MPTP)), and ischemic brain damage models [51]. The proposed in vitro cellular models and in vivo animal models have shed light on the molecular mechanisms underlying oxidative stress responses in the nervous system, such as cell survival and cell death. Among neurotoxic chemical agents, 6-hydroxydopamine (6-OHDA) has been used to induce neurotoxicity in the dopaminergic nigrostriatal system by inhibiting the mitochondrial electron transport chain of complexes I and IV and accelerating neuronal degeneration [64,65,66].

6-OHDA has been regarded as an endogenous toxic factor in the pathogenesis of PD. The neurotoxin 6-OHDA induces excessive production and accumulation of ROS and, therefore, oxidative stress. In fact, 6-OHDA induces caspase-3 activation in the cells mediated by the Fas or mitochondrial pathways [67]. Indeed, it has been demonstrated that MTPT/6-OHDA-induced NF-κB activation in SH-SY5Y neuroblastoma cells triggers caspase-3 activation, which results in the death of DCNs via the NF-κB pathway [68,69,70].

In vitro studies have evaluated the toxic effects of 6-OHDA in dopaminergic (DArgic) cell cultures. For instance, Vestuto et al. used human neuroblastoma SH-SY5Y cells to assess the neuroprotective effect of cocoa extract (purified fractions) in a 6-OHDA-induced PD cellular model [71]. Similarly, Chansiw et al. reported the protective effect of 1-(*N*-acetyl-6-aminohexyl)-3-hydroxy-2-methyl pyridine-4-one (CM1) coupled with green tea extract (GTE) on iron-induced oxidative stress in SH-SY5Y cells [72]. In a separate study, Chen et al. evaluated the protective effect of EGCG against 6-OHDA-induced neurotoxicity by using N27 dopaminergic neurons [73].

The advantages of cellular systems for studying oxidative stress are their low cost, adaptability, modularity, reproducibility, compatibility with high-throughput screening, and interest in cell mechanical investigations without systemic interferences. Other studies have demonstrated that hypoxia can increase cells’ susceptibility to oxidative stress. The hypoxia-reoxygenation model is a relevant in vitro model of oxidative stress, given that cellular hypoxia appears to be a crucial signal that activates transcriptional regulators, specifically hypoxia-inducible factor-1 (HIF-1) [74], nuclear factor kappa B (NF-κB) [75], activator protein 1 (AP-1) [76,77], and some mitogen-activated protein kinase (MAPK) signaling pathways, and induces cell death and necrosis [76,77].

Genetically derived models of neurodegenerative diseases are gaining considerable interest because they are excellent surrogates, providing intrinsic validity to genetically based models of degenerative disorders [78]. Scientific investigations have reported that knockout of genes, including PINK1, DJ-1, LRRK2, and LRRK1, in rats leads to age-dependent neurodegeneration of the dopaminergic neurons of PD [78,79,80]. Moreover, mutation in the copper-zinc superoxide dismutase 1 (SOD1) gene has been associated with ALS, while the alteration of MAPT genes or progranulin is linked to frontotemporal dementia (FTD) [81]. Other studies have highlighted that mutations in amyloid precursor protein (APP), presenilin 1, and presenilin 2 (PSEN1/2) are the main causes of autosomal dominant early-onset AD [82,83].

It has been documented that invertebrates can also mimic endogenous-generated ROS. Such a model has been widely implemented in Drosophila melanogaster and *Caenorhabditis elegans* (*C. elegans*) [84,85]. The initial longevity mutant, known as age-1, which contains increased levels of both SOD1 and catalase, is arguably the best-studied mutant in the nematode *C. elegans*. The age-1 mutant exhibits more significant levels of both SOD1 and catalase enzymes because this gene, which encodes for phosphatidylinositol-3 kinase, confers a longer lifetime phenotype when silenced, along with improved resilience to several forms of stress [85,86,87].

The aforementioned findings indicate that oxidative-stress-based pharmaceutical therapies can slow aging and degeneration, as summarized in Table 1. Therefore, the developed in vivo and in vitro models of oxidative stress can be of interest for evaluating the efficacy of nanotherapeutic management of long COVID conditions.

## 3. Ginkgo Biloba Extract (EGb) for Neuroprotection and Potential Regeneration from Long COVID Syndrome

### 3.1. Ginkgo Biloba Antioxidative and Anti-Inflammatory Effects

Ginkgo biloba (GB) is one of the medicinal plants that ameliorate capillary blood circulation, provide brain oxygenation, and thus improve age-related disorders. The ginkgo tree is monotypic and belongs to the class Ginkgoopsida, considered the oldest tree alive in the world (ginkgo species are from the Permian period, around 286–248 million years ago) [23]. The currently available herbal medicines based on Ginkgo biloba extract (EGb) are Tebonin^®^ and Tanakan^®^, which are mostly standardized on ginkgo flavone glycosides and terpene lactones—EGb 761^®^ [26]. The standardized extract of Ginkgo biloba leaves includes 6% terpenoids, of which 3.1% are ginkgolides A, B, C, and J and 2.9% are bilobalide. It contains 24% flavonoid glycosides, including quercetin, kaempferol, and isorhamnetin, and 5–10% organic acids (Figure 2) [100]. Ginkgo biloba leaf extract is mentioned in the *British Herbal Compendium* as a treatment for mild-to-severe dementia, including Alzheimer’s disease, and for the treatment of neurological symptoms attributed to loss of concentration and poor memory, confusion, depression, anxiety, vertigo, tinnitus, and headache [101].

The neuroprotective effect of EGb 761 extract has been examined in a rat model of cerebral injury following ischemia/reperfusion (I/R). The treatment resulted in a decrease in MDA levels, the downregulation of pro-inflammatory cytokines (TNF-α and IL-1β), and an increase in the expression of anti-inflammatory cytokines (IL-10) and enzymatic SOD and myeloperoxidase (MPO) activities, which can control neurological impairments [102]. It can be suggested that the beneficial effects of EGb on cerebral ischemia/reperfusion I/R injury result from the reduction in oxidative stress due to the inhibition of nitric oxide production and inflammation induced by I/R [102].

Kaempferol is one of the most important constituents of Ginkgo biloba, and its action accounts for the upregulation of the glutamate-cysteine ligase catalytic (GCLC) subunit, brain-derived neurotrophic factor (BDNF), B-cell lymphoma protein 2 (Bcl-2), and GSH [103,104,105]. Kaempferol inhibits ROS generation by scavenging free radicals and efficiently protects neuronal cells from oxidative injury. Additionally, it inhibits the production of pro-apoptotic proteins, including Bax and caspase-3, and modulates the downregulation of the NF-κB pathway to exert anti-apoptotic effects [106]. A study by Zhou et al. showed that kaempferol can inhibit mitochondrial membrane transition (mPTP) opening and suppresses the release of cytochrome C via GSK-3β inhibition [107]. Kaempferol is also involved in the inhibition of serotonin breakdown by monoamine oxidase, reduces neurotoxicity induced by 3-nitropropionic acid (3-NP), and induces the upregulation of heme oxygenase 1 (HMOX-1) [108].

Quercetin, bilobalide, and isorhamnetin are other essential compounds in Ginkgo biloba extracts. Bilobalide decreases the expression of reactive species induced through H_2_O_2_, thus inhibiting ER stress [109]. It can also suppress pro-inflammatory activation, NF-κB, and COX-2 activities [110]. Studies have reported the beneficial effects of bilobalide in the upregulation of c-myc and p53 proteins, inhibition of the degradation of membrane phospholipids, and increased cellular proliferation of neurons in the hippocampus [109,111,112]. According to Wang et al., pretreatment with bilobalide substantially reduced COX-2, iNOS, and phosphorylated p65 in sepsis-induced CLP mouse models, while inducing I-kB activation in the lungs [111]. Additionally, bilobalide reduced oxidative stress by increasing HO-1 expression in lung tissues and antioxidative enzyme genes, including catalase, MnSOD, CuZnSOD, and GPx-1 [111]. Moreover, bilobalide and EGb50 can modulate the expression of TLR4, NF-B, and MyD88, preventing the onset of acute lung injury (ALI) [111,113,114]. Ginkgo biloba components may therefore prevent the onset of ALI and the cytokine storm syndrome in COVID-19 by inhibiting pro-inflammatory signaling via the NF-κB and TLR4 signaling pathways. A study showed that the administration of EGb50 substantially lowers TNF- and IL-1 levels and prevents the related signal transduction through the p38 MAPK and NF-B p65 pathways in LPS-stimulated microglial cells [115]. In a separate study, EGb50 demonstrated a potential anti-inflammatory action by suppressing NLRP3-inflammasome-induced microglial activation [116]. For comparison, isorhamnetin has been linked with the inhibition of apoptosis and the suppression of DNA fragmentation [117].

In an enzymatic inhibition assay, it was established that ginkgolide A can act as an irreversible inhibitor against SARS-CoV-2 papain-like protease (PLpro) at a nontoxic dose of 1.79 µM [20]. Similarly, quercetin, the primary EGb flavonoid component, inhibits SARS-CoV-2 3-chymotrypsin-like protease (3CLpro) and PLpro, with a corresponding docking energy of 6.25–4.62 kcal/mol, preventing SARS-CoV-2 replication [118].

In a recent study, Liu et al. compared the antioxidant capacity of various Ginkgo biloba extracts by evaluating the mechanisms of ginkgolides A (GA), B (GB), K (GK), and bilobalide (BB) against oxidative stress caused by transient focal cerebral ischemia [119].

**Figure 4:**
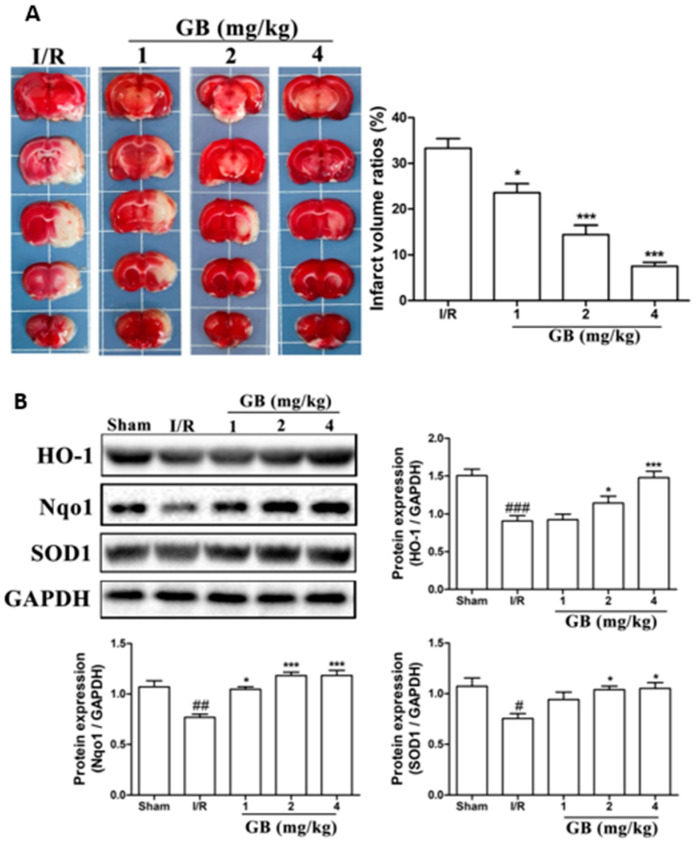
Effects of Ginkgo biloba on infarct volume and expression of proteins associated with antioxidant effects in middle cerebral artery occlusion (MCAO) rat models. (**A**) MCAO rats were treated with various GB doses for 72 h, followed by triphenyl tetrazolium chloride (TTC) staining and statistical analysis of the cerebral infarct area. The results revealed a significant reduction in infarct volume ratios after GB treatments in a dose-dependent manner. (**B**) Results of Western blot analysis and semi-quantitative measurements of the levels of HO-1, Nqo1, and SOD1 proteins in the ischemic penumbra region of MCAO rats treated with various doses of Ginkgo biloba for 24 h. Antioxidant-related proteins in the MCAO group drastically decreased after cerebral ischemic injury compared to the normal group, while rats treated with various concentrations of GB showed a marked increase in the expression of HO-1, Nqo1, and SOD1. Data represented as the mean ± SD from eight rats of each group. (# *p* < 0.05, ## *p* < 0.01, and ### *p* < 0.001 vs. the sham group; * *p* < 0.05 and *** *p* < 0.001 vs. the I/R group). Adapted with permission from [119]. Copyright {2019} Science-HHS Public Access (PubMedCentral).

In vivo studies have been performed in a developed middle cerebral artery occlusion (MCAO) model of cerebral ischemic injury using male SD rats, followed by reperfusion and Ginkgo biloba treatments [119]. Neuroblastoma cells (SH-SY5Y) were subjected to oxygen-glucose deprivation (OGD) for 4 h, followed by 6 h of reoxygenation using ginkgolides and bilobalide. The in vitro experimental findings revealed that GA, GB, GK, and BB significantly reduce ROS and increase SOD activities and protein levels, including HO-1 and Nqo1. Additionally, p-Akt and p-Nrf2 levels considerably increased following ginkgolide and BB treatments, with GB demonstrating greater efficacy than GA and GK. These upregulations could be reduced in a dose-dependent manner by LY294002, a PI3K inhibitor. The triphenyl tetrazolium chloride (TTC) staining performed demonstrated that GB significantly reduced the infarct volume ratios in MCAO rats in a dose-dependent manner (Figure 4a). Additionally, GB markedly increased the amounts of the proteins HO-1, Nqo1, SOD, p-Akt, p-Nrf2, and Nrf2 through the modulation of the Akt/Nrf2 signaling pathway, shielding neurons from oxidative-stress-related damage (Figure 4b) [119].

Table 2 summarizes the chemical structures of the active constituents identified in Ginkgo biloba extracts and their reported biological activities of potential interest to recovery from neurological long COVID syndrome.

### 3.2. Neuroprotective, Anti-Apoptotic, and Anxiolytic Drug Effects of EGb

Results from microarray experiments have established the neuroprotective effect of Egb 761 against ischemic-induced neuronal injury [139]. The data revealed that the upregulation of Bcl-2 protein may be mediated by the activation of cAMP-response-element-binding protein (CREB) [139]. EGb 761 increases CREB phosphorylation via the activation of PI3K/Akt and extracellular-signal-regulated kinase (ERK) signaling pathways [140]. The consequently released BNDF protects the neurons against ischemia. Moreover, Tchantchou et al. affirmed that EGb 761 can reduce Aβ oligomerization and promote neurogenesis by the phosphorylation of CREB. This was evidenced by enhanced cell proliferation in the hippocampus of TgAPP/PS1 mice [141]. Overall, these studies have demonstrated that flavonoids, the primary active constituents of EGb 761, may upregulate the CREB–BDNF pathway and therefore exert neuroprotection.

Combination therapy of EGb 761 with bone-marrow-derived mesenchymal stem cells (BMSCs) has shown a synergistic effect in animals with autoimmune encephalomyelitis. The therapeutic mechanism involves the inhibition of pro-inflammatory cytokines, demyelination, and protection axons and neurons [142]. Other studies have documented that GA prevents p-Tau deposition and thus protects cells from toxicity associated with Tau hyperphosphorylation. Interestingly, the degree of dementia is closely correlated with the production of hyperphosphorylated Tau aggregates, making EGb 761 crucial to counteract the neurodegenerative process [143].

Ginkgo biloba extracts, mainly flavonoids and ginkgolides, exert inhibitory effects on acetylcholinesterase activity. In fact, cholinergic agonists can reduce inflammation by blocking inflammatory signals, particularly the ubiquitous nuclear protein HMGB1 that is released by dying cells or activated innate immunity cells to promote inflammation [144]. It has been suggested that nicotinic receptors nAChRs may control the expression of ACE2 and serve as a binding receptor for S1 protein, leading to an inflammatory response. However, EGb may counteract the central inhibitory and anti-inflammatory effects of GABAergic neurons, leading to increased cortical neuronal activity and an increased risk of convulsion [145,146]. However, meta-analysis research has proven that there is no convulsion risk associated with the anxiolytic action of EGb, which is mediated through the regulation of GABAergic neurons [147]. In patients with dementia, EGb 761^®^ has shown promise in alleviating comorbid neurosensory symptoms and improving memory deficits [147].

**Figure 5:**
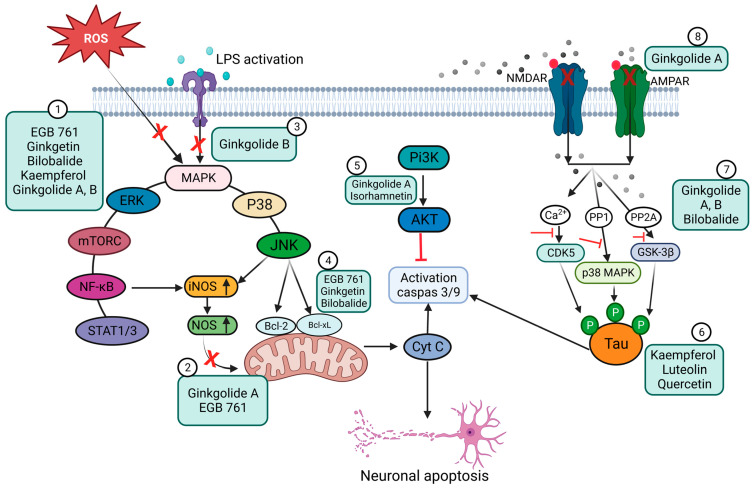
Schematic representation of the anti-apoptotic and anti-inflammatory effects of Ginkgo biloba extract (EGb) and its constituents. (1) Inhibition of ROS and suppression of the expression of pro-inflammatory mediators (e.g., COX-2 and NO) and pro-inflammatory cytokines (TNF-α, IL-6, and IL-1β) via the NF-κB signaling pathway. EGb can also inhibit the STAT 1/3 pathway. (2) Blocking of iNOS expression through a reduction in NO levels. (3) Inhibition of LPS-induced inflammatory response. (4) Prevention of mitochondrial oxidative stress by promoting the expression of anti-apoptotic proteins. (5) Inhibition of TLR4-NF-κB signaling through the PI3K/Akt pathway. (6) Prevention of the intracellular accumulation of p-Tau and cellular protection from Tau-hyperphosphorylation-related toxicity. (7) Blocking signaling pathways that involve CDK5, p38 MAPK, and GSK-3β. (8) Inhibition of NMDA and AMPA receptors, preventing the phosphorylation of c-Jun N-terminal kinase (JNK). (Created with BioRender).

In another study, EGb 761 showed neuroprotective effects against oxidative-stress-induced apoptotic cell death by inhibiting apoptosis in a p53-dependent pathway, preventing mitochondrial membrane damage, reducing the release of cytochrome C from the mitochondria, upregulating the anti-apoptotic protein Bcl-2, and inhibiting PARP cleavage [148]. The anti-apoptotic effects are schematically presented in Figure 5.

The work of Wang et al. showed that EGb may be used to increase cerebral blood flow and cognitive function by co-administration with 75 mg of aspirin toward the treatment of vascular cognitive impairment of non-dementia [149]. In a randomized, double-blind exploratory study, the authors demonstrated that administration of EGb (Symfona^®^ forte) at a dose of 120 mg/twice daily for at least 6 months may improve dual-task-related gait performance in patients with MCI [150]. Moreover, extensive work by Kuo et al. found that GA exhibits a strong therapeutic promise, like memantine, for treating AD by blocking NMDA and AMPA receptors. GA also suppresses c-Jun N-terminal kinase (JNK) activation at different doses (1–200 μM) in Aβ-induced neuronal depolarization in mice (Figure 5) [151].

**Figure 6:**
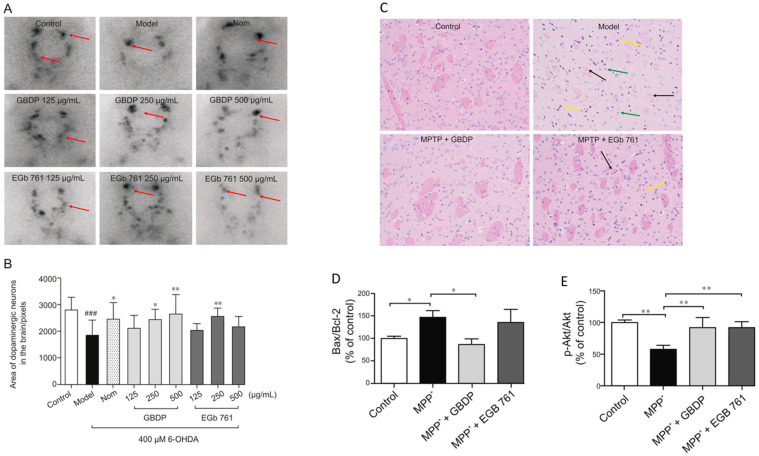
Representative images of dopaminergic neurons in the zebrafish brain, acquired using TH immunostaining. (**A**) Ginkgo biloba dropping pills (GBDP) prevented the loss of dopaminergic neurons induced by 6-OHDA. The red arrow shows dopaminergic neurons in the zebrafish brain. (**B**) The area of the dopaminergic neurons calculated for each group. ### *p* < 0.0001 vs. the control group; * *p* < 0.05 and ** *p* < 0.001 vs. the 6-OHDA group (*n* = 10 per group). (**C**) HE staining of brain sections of an MPTP-induced mouse model of Parkinson’s disease. The black arrow indicates significant diminished and loose nerve fiber components, which are lightly stained, while the yellow arrow shows intensive staining of the nuclei of several atrophied cells. Glial cells exhibited modest hyperplasia, as shown by the green arrow. (**D**,**E**) In MPP-treated human SH-SY5Y cells, GBDP administration reduced the Bax/Bcl-2 ratio and elevated Akt/GSK3β. ### *p* < 0.0001 vs. the MPP+ group. Adapted with permission from [152]. Copyright {2021} Science-HHS Public Access (PubMedCentral).

In parallel, Yu et al. demonstrated the neuroprotective effect of Ginkgo biloba dropping pills (GBDP) in the amelioration of PD [152]. In that study, the pharmacological effects of GBDP and EGb 761 were exploited in both in vivo and in vitro models of PD. Following GBDP and EGb 761 treatments, the viability of DA neurons in zebrafish was assessed via tyrosine hydroxylase immunostaining. Dopaminergic neurons in zebrafish were significantly lost after exposure to 400 mM 6-OHDA for 48 h. Nevertheless, administration of 250 or 500 mg/mL of GBDP or 250 mg/mL of EGb 761 rescued the death of dopaminergic neurons induced with 6-OHDA. No protection was observed with further GBDP and EGb 761 dosages (Figure 6A,B). Moreover, GBDP reduced cognitive impairment and neuronal damage in MPTP-induced PD mice and reversed the effect of 6-OHDA-induced dopaminergic neuronal loss in zebrafish (Figure 6C). In vitro findings revealed that the neuroprotective effects of GBDP can be mediated through the Akt/GSK3β pathway (Figure 6D,E) [152].

Table 3 summarizes the reported neuroprotective effects of EGb.

### 3.3. Bioavailability and Safety of EGb

Acute toxicity studies have shown that EGb has a lethal dose (LD50) of 1.1 g/kg, 1.9 g/kg, and 7.73 g/kg in mice and 1.1 g/kg, 2.1 g/kg, and >10 g/kg in rats when administered intravenously, intraperitoneally, and orally, respectively [168,169]. In the older population, EGb is frequently used in the management of type 2 diabetes (T2DM), hypertension, or rheumatism. However, the interaction of EGb with other drug candidates can lead to bleeding. In a retrospective case series study, a 73-year-old man who had been taking 75 mg of a ginkgo supplement for 6 months as an aid to improve his memory deficits experienced episodes of spontaneous bleeding. The supplement included 27% ginkgo flavone glycosides and 10% terpene lactones [170]. A case report revealed that a 38-year-old lady who had been taking thiamine and Ginkgo biloba extract (240 mg/day) for 4 years experienced a brain hemorrhage [171]. Another study documented the case of a 70-year-old man with spontaneous iris bleeding (hyphema). This clinical occurrence took place 1 week after the patient started taking Ginkoba, a different natural Ginkgo biloba supplement (40 mg twice a day), in combination with 325 mg of aspirin every day. Ginkoba was withdrawn from therapy after monitoring the case for 3 months, and no further bleeding incidents were observed. Thus, it was proposed that the bleeding was caused by interactions between aspirin and gingko biloba extracts [172].

From the examples cited, research connecting Ginkgo biloba therapy with bleeding risk includes case reports. In addition, comprehensive analyses of randomized controlled studies have not identified any elevated risk of bleeding in patients using Ginkgo biloba extracts [173,174]. For example, in a prospective, double-blind, randomized, placebo-controlled trial, the administration of EGb 761 to young, healthy male volunteers at three different doses (120, 240, and 480 mg/day) had no effect on platelet function or coagulation [175]. Moreover, it has been reported that EGb 761 at a dosage of 120 mg/day inhibits platelet aggregation and thromboxane B2 synthesis [176]. In general, there is inconclusive evidence that Ginkgo biloba extracts are associated with bleeding. However, further research is necessary to resolve these controversies.

## 4. Perspectives for Use of Ginkgo Biloba in Nanotherapies of Neurological Disorders

**Figure 7:**
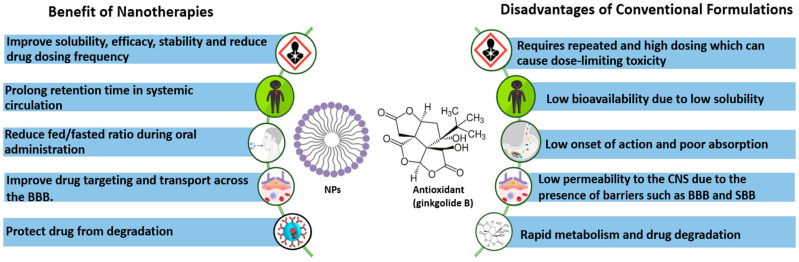
Advantages of nanotherapies over conventional formulations [183,184,185].

Although preclinical studies of antioxidants to improve neuronal dysfunction have shown encouraging results, the outcomes of clinical trials have not always been conclusive. In general, antioxidant compounds have mainly suppressed clinical symptoms but are unable to halt or reverse disease progression [177]. In the absence of delivery systems, Ginkgo biloba (120 mg daily dosage) did not improve patient performance in neuropsychological tests of memory, attention, or speech during a 6-week, placebo-controlled, double-blind clinical study including 219 individuals [178]. Similarly, a feasibility investigation by Dodge et al. reported no difference in episodic memory deterioration in patients who received 240 mg/day of Ginkgo biloba or a placebo during an average follow-up period of 3.5 years [179]. The clinical translation of free antioxidant drugs may be hampered by several issues related to drug delivery efficacy. These problems include low drug bioavailability, low permeability through the CNS, limited half-life, and toxicity [180] (Figure 7). Significant efforts have been made to increase the clinical efficacy of both natural and synthetic antioxidants by using drug delivery systems to overcome their drawbacks. For example, investigations of drug conjugates, complexes, and nanocarriers of various polymeric materials have been performed [181,182,183].

### 4.1. Intranasal Administration and Biodistribution of Nanoparticulate Carriers

Delivering nanomedicines to the brain to treat CNS disorders is a major advantage of circumventing the BBB and reducing systemic exposure. Intranasal administration has attracted attention as a potential delivery mechanism to the brain for neuroprotection. The intranasal delivery method has been proposed for the treatment of CNS disorders (e.g., migraine, sleep disorders, brain tumors, multiple sclerosis, PD, and AD), thanks to its non-invasiveness and high patient compliance [184,186,187,188]. Because the olfactory mucosa is in direct contact with the CNS, intranasal delivery of small and large molecules can successfully target the brain. This prevents the accumulation of drug molecules in vital organs, such as the liver, spleen, and kidney, which would lessen systemic adverse effects. Alternative delivery routes are via the lymphatic and vascular systems [189,190].

Nanotechnology has considerable potential for delivering therapeutic compounds to the brain through different mechanisms that allow blood–brain barrier passage. Nanoparticles (NPs) can improve drug solubility, increase the residence time of the active compounds at the target site, enhance their mucosal permeation and cellular internalization, regulate the release of encapsulated drugs, and lessen systemic side effects by limiting the distribution to non-targeted areas [189].

**Figure 8:**
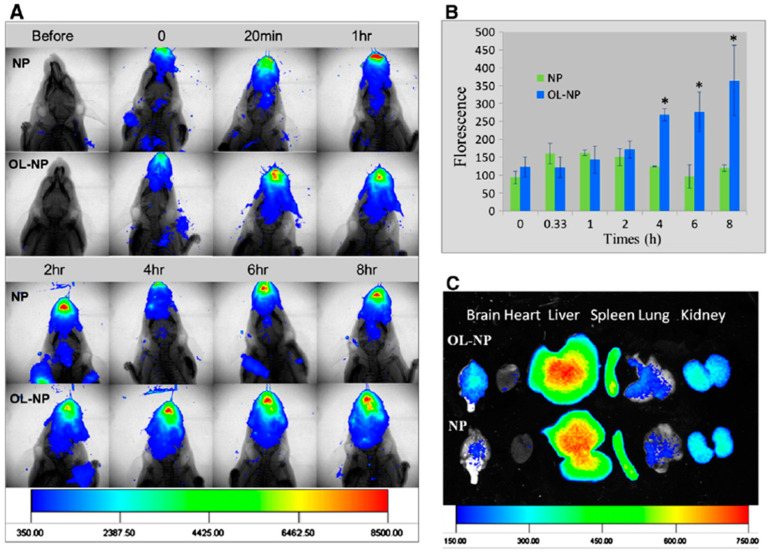
Delivery of odorranalectin (OL)-conjugated NPs to the brain monitored with an in vivo imaging system. (**A**) Fluorescence images of the upper half-body of the mouse overlaid on X-ray images, following intranasal administration of DiR-labeled NPs and OL-conjugated NPs at different time points. (**B**) Semi-quantitative results of the fluorescence intensity in the brain region. * *p* < 0.05, significantly different. (**C**) Fluorescence images of major organs overlaid on white-light images at 8 h after intranasal administration of DiR-loaded NPs and OL-conjugated NPs to mice. Copyright © 2011 Elsevier B.V. All rights reserved, with permission from Elsevier [191].

Wen et al. proposed nasal nanotherapy targeted to the brain with decreased immunogenicity with a drug delivery system. PEG–PLGA nanoparticles were modified with conventional lectin by combining a synthetic OL–conjugate PEG–PLGA (OL–PEG–PLGA) with PEG–PLGA [191]. For nanotherapy, odorranalectin (OL)-modified NPs were fabricated via the double-emulsion technique. A hemagglutination test was conducted to validate the biorecognitive activity of OL on the surface of the NPs. The results in Figure 8 show that OL-conjugated NPs possess hemagglutinating activity and demonstrate improved bioactivity. The nose-to-brain transport properties of the OL-conjugated NPs were examined using an in vivo fluorescent imaging approach with DiR dye as a tracer. The nanocarriers were co-administered with urocortin peptide via the intranasal route. The therapeutic efficacy of hemiparkinsonian rats was assessed using a rotation behavior test, a tyrosine hydroxylase test, and a neurotransmitter determination test. The obtained data suggested that the intranasal delivery of functionalized NPs to the brain enhances the therapeutic efficacy of nanodrugs in PD models [191].

### 4.2. Ginkgo-Biloba-Based Nanotherapy for Neuroprotection and Regeneration from SARS-CoV-2 Neurological Damage 

In a recent study, Ginkgo-biloba-extract-loaded chitosan nanoparticles (Gb–CsNPs) were synthesized via an ionic gelation method. The outcomes revealed that the average size of the Gb–CsNPs was 104.4 nm, with a zeta potential of 29.3 mV and a polydispersity index (PDI) of 0.09. The encapsulation efficacy and drug-loading capacity were 40% and 97.4%, respectively. The neuroprotective efficacy of the Gb–CsNPs was examined in an oxidative-stress-induced cellular model (SH-SY5Y). The results showed increased cell survival from 60% to 92.3%, proving the NPs’ efficacy and biocompatibility [192]. Additionally, the encapsulation of EGb in chitosan NPs improved its neuroprotective properties [192].

Wang et al. developed Ginkgo biloba extract nanoparticles to enhance the oral bioavailability of GBE in Sprague–Dawley rats at a dosage of 40 mg kg^−1^. The Cmax value of the flavonoids in raw GBE and GBE nanoparticles was reported to be 2.949 lg mL^−1^ at 0.5 h and 4.302 lg mL^−1^ in 0.333 h, respectively [193]. In a separate study, Zhao et al. demonstrated the ability of GBE NPs to transport across barriers, including the chorion, the GI barrier, the BRB, and the BBB. GBE was encapsulated by using poly(ethylene glycol)-co-poly(ε-caprolactone) (PEG–PCL) nanoparticles. The developed nanoparticles facilitated the sustained release and enhanced brain uptake of GBE in the plasma of treated animals to treat PD [194].

Additionally, the delivery of quercetin across the BBB was achieved by using SLNs via intravenous administration to improve the therapeutic efficacy of this molecule [195]. The high-pressure homogenization procedure was used to successfully formulate SLNs loaded with Ginkgo biloba extract. The SLNs exhibited an appropriate particle size and shape, sustained the release profile, and improved the loading efficiency of the active substance [196].

In contrast, Xu et al. used an aqueous extract of Ginkgo biloba leaves to synthesize AgNPs with a mean particle size of 40.2 ± 1.2 nm, a polydispersity index of 0.091 ± 0.011, and a zeta potential of −34.56 mV [197]. In vitro results showed that EGb–AgNP treatment significantly increases intracellular ROS levels, facilitates cytochrome C release from the mitochondria into the cytosol, and facilitates caspase-9 and caspase-3 cleavage. This indicated that EGb–AgNPs can induce the activation of caspase-dependent mitochondrial apoptotic pathways, which are significant for various therapeutic applications [197].

**Figure 9:**
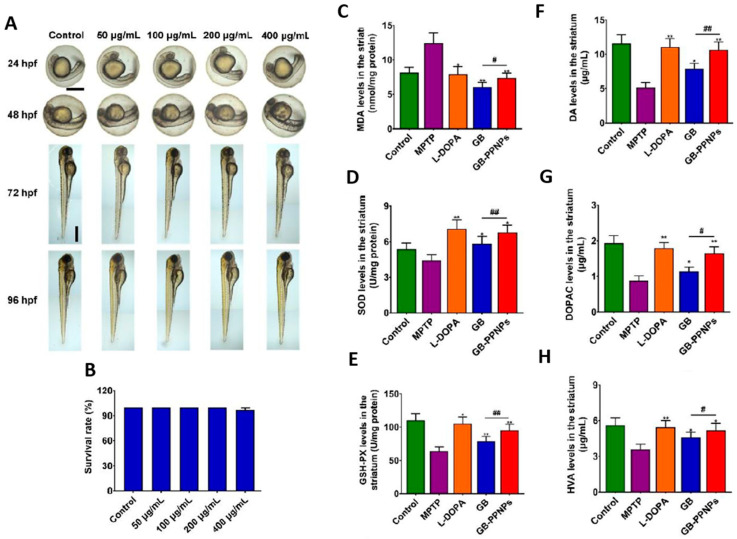
In vivo toxicity analysis of ginkgolide-B-loaded GB–PPNPs in zebrafish embryo and MPTP-induced murine model of PD. (**A**) Zebrafish embryos were treated with different concentrations of GB–PPNPs (50, 100, 200, and 400 μg/mL), and at 96 hpf, the embryo morphology was visualized via microscopy. The survival rates, hatching rates, heart rates, and zebrafish body length were calculated. Scale bar: 500 μm. (**C**–**H**) Impact of GB–PPNPs on the striatum with determined levels of (**A**) MDA, (**B**) SOD, (**C**) GSH-Px (means ± SD, *n* = 4), (**D**) dopamine, (**E**) DOPAC, and (**F**) HVA (*n* = 7). * *p* < 0.05 and ** *p* < 0.01 correspond to different treatments vs. MPTP. # *p* < 0.05 and ## *p* < 0.01 correspond to GB–PPNPs vs. GB. Adapted with permission from [198]. Copyright {2022} Science-HHS Public Access (PubMedCentral).

As a potential nanotherapy for Parkinson’s disease (PD), Wang et al. synthesized biodegradable poly(ethylene glycol)-b-poly(trimethylene carbonate) nanoparticles (PPNPs) to deliver ginkgolide B. This enhanced the accumulation of bioactive molecules in the blood and brain [198]. The fabricated GB–PPNPs effectively promoted the sustained release of ginkgolide B for 48 h. Moreover, the GB–PPNPs at various concentrations (50, 100, 200, and 400 µg/mL) prevented the neurotoxicity induced by MPP+ and protected zebrafish embryos or larvae, while decreasing the level of MDA protein expression in GB–PPNP-treated mice compared to MPTP-treated mice (Figure 9). Further research revealed that mice treated with GB–PPNPs had higher levels of SOD and GSH-Px than mice treated with MPTP. Additionally, GB–PPNPs elevated the concentration of DOPAC (10.66 ± 1.12, 1.65 ± 0.18 µg/g) and HVA (5.17 ± 0.60 µg/g). These values were significantly higher than those observed in the disease group [198].

### 4.3. Green Synthesis of Ginkgo Biloba nanoEGb

**Figure 10:**
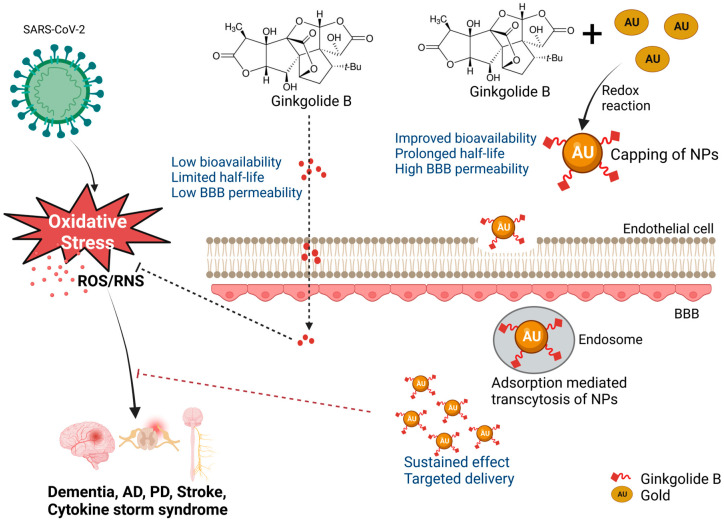
Antioxidant-based nanotherapy via green synthesis of nanoparticles. The scheme illustrates the benefits of delivering Ginkgo biloba biogenic metallic NPs to increase their half-life, capacity to penetrate the BBB, bioavailability, and sustained effect, thus neutralizing oxidative stress in neurological disorders (created with BioRender).

Ginkgo biloba is synthesized as nanoEGb using a variety of nanocarriers, such as liposomes, polymers, cyclodextrins, micelles, and carbon-based nanoconjugates, to address its limited water solubility and poor bioavailability and improve its half-life and retention time (Table 4) [199]. The poor bioavailability of EGb has been associated with the presence of diterpenoid molecules, particularly in ginkgolides A, B, and C [199]. The biosynthesis of Ginkgo biloba leaves has been realized using gold nanoparticles (AuNPs), Fe_3_O_4_ magnetic nanoparticles (MNPs), and silver nanoparticles (AgNPs) [197,200,201]. This technique was demonstrated by Elshazly et al., who developed AgNPs using Ginkgo biloba extract. The nanoparticles exhibited a mean particle size ranging from 5.46 to 19.40 nm and an average diameter of 11.81 nm. In vitro experiments demonstrated that AgNPs have a moderate inhibition against MERS-CoV and HCoV-229E, which share similar sequence homology with SARS-CoV-2 [202]. Figure 10 demonstrates the potential uptake mechanism of an EGb nano-conjugate via transcytosis.

### 4.4. Characterization Techniques for Nanotherapeutics

For the development of nanotherapeutics, nanoparticles are characterized using different techniques [198,199,200,201,202,203,204,205,206,207,208,209,210,211,212,213]. Sufficient knowledge of the safety, efficacy, and quality of nanotherapies is required to enable easy translation toward clinical applications [211]. Various techniques are used to characterize the size, charge, morphology, drug encapsulation efficiency, drug loading, toxicity, etc., including dynamic light scattering (DLS), nanoparticle tracking analysis (NTA), UV–VIS spectrometry, transmission electron microscopy (TEM), scanning electron microscopy (SEM), and cytotoxicity assessment via (3-(4,5-dimethylthiazol-2-yl)-2,5-diphenyltetrazolium bromide (MTT) and lactate dehydrogenase (LDH) assays (Table 5) [212,213].

## 5. Conclusions

It is indispensable to continue studying the mechanisms that underlie the pathophysiological process of SARS-CoV-2 infection. This will enable researchers to uncover the therapeutic targets that may be used for their management. According to this review, it may be suggested that Ginkgo biloba has potential positive effects, including anxiolytic, antineurotoxic, anti-inflammatory and anti-apoptotic functions, and has been explored in treating neurological disorders, particularly AD, PD, and dementia. Nevertheless, further studies are needed to corroborate the activity and mechanisms of action of this phytochemical since it could constitute an alternative for the treatment of vascular and degenerative diseases. Nanotechnology-based drug delivery systems could be an approach to address the limitations of antioxidant compounds, which include insufficient dosing, limited bioavailability, restricted transport to the CNS, transient retention, and low antioxidant capacity to completely scavenge the effect of ROS. The development of experimental techniques to mimic ROS has made it possible to study oxidative stress in the CNS. These methods will be fundamental for future discoveries related to the role of oxidative stress in neurological diseases.

## Figures and Tables

**Table 1:** Stimuli that trigger oxidative stress in cells or in the CNS, similar to the SARS-CoV-2 action.

Stimuli	Model/Species	Disease Model	Administration/Protocol	Mechanism of Oxidative Stress	Ref.
*N*-methyl- 4-phenyl-1,2,3,6-tetrahydropyridine (MPTP)	Male C57BL/6 mice (20–25 g)	PD, neurodegeneration	Intraperitoneal injection of MPTP (20 mg/kg), two times at 4 h intervals daily for 5 days, followed by oral administration of Sophora tomentosa (25 mg/kg, 50 mg/kg, and 100 mg/kg) for 15 consecutive days until behavioral tests	MPTP is taken up by astrocytes or serotonergic neurons, where it is metabolized into 1-methyl-4-pyridinium (MPP+) by monoamine oxidase B. Subsequent uptake of MPP+ via dopamine, serotonin, and norepinephrine transporters induces oxidative stress by inhibiting complex I of the respiratory chain. Selectively destroys the nigrostriatal dopaminergic pathway and thus is widely used as a PD model. Upregulates levels of MDA, α-synuclein overexpression, and GSK-3β phosphorylation in the mouse striatum.	[88]
Rotenone	Rotenone-induced Sprague–Dawley and Lewis rats	PD	Infusion of a 2–3 mg/kg dose of rotenone per day via a jugular vein cannula attached to a subcutaneous osmotic minipump	Inhibits of complex I and degenerates the nigrostriatal dopaminergic pathway associated with hypokinesia and rigidity.	[89]
Paraquat (*N*, *N*′-dimethyl-4-4′-bipiridinium)	Human neuroblastoma SH-SY5Y cells	PD	Treated with paraquat (0.5 mM PQ) for 48 h	Increases superoxide levels and neuronal cell death. Decreases dopamine levels in the substantia nigra and increases α-synuclein expression. Decreases protein levels of Nrf2, γGCS levels, and intracellular GSH levels.	[90]
Hydrogen peroxide (H_2_O_2_)	SH-SY5Y cells	PD, AD, Huntington’s disease	Incubation with varying concentrations of H_2_O_2_ (0 to 250 µM) for 30 min, followed by evaluation of cell viability	Loss of viability <5% at concentrations up to 250 µM H_2_O_2_. Cell membrane and DNA damage accompanied by decreased SOD activity but increased GPX activity in cells treated with >50 µM concentration of H_2_O_2_.	[91,92]
6-hydroxydopamine (6-OHDA)	SH-SY5Y cells	PD, AD, and dementia	Cells incubated with 200 µM of 6-OHDA for 24 h with or without hyperoxide or NAC pretreatment	Inhibits both complexes I and IV of the respiratory chain and leads to the generation of superoxide, hydrogen peroxide, and hydroxyl radicals.	[93]
Glutamate analog; homocysteate quisqualate ibotenate	Neuronal hybridoma cell line, N18-RE-105 mouse neuroblastoma cells	ALS, AD, dementia, PD, multiple sclerosis (MS)	Continuous exposure of cells to ʟ-glutamate (1–10 mM) or quisqualate (0.1–1.0 mM) for 5 min	Induces cytotoxicity by inhibiting cystine uptake and resulting in lowered glutathione levels, leading to oxidative stress and cell death.	[94,95]
Mycotoxin 3-nitropropionic acid (3-NP)	Male Wistar rats (300–350 g)	Huntington disease	Intraperitoneal administration of 3-NP (10 mg/kg)	Irreversibly inhibits the Krebs cycle and complex II of the respiratory chain. Promotes the generation of hydroxyl radicals, leading to neuronal death. Induces motor dysfunction.	[96]
Buthionine sulfoximine (BSO)	Hippocampus-derived immortalized cell line (HT22)	Chronic psychological stress	Treatment with 1 mM BSO for 14 h	Binds to glutathione synthetase to inhibit glutathione production.	[97]
Tunicamycin	SH-SY5Y cells	Endoplasmic reticulum stress	Incubation with 1 μM tunicamycin	Intracellular accumulation of aggregates of misfolded protein.	[98]
RNAi	Drosophila	Oxidative damage	Knockdown of SOD2 using the Gal4/UAS system to express SOD2 inverted repeat (*Sod2*-IR) transgenes	Degrades mRNA related to the expression of a specific antioxidant. Increases caspase activity, decreases mitochondrial content, and reduces ATP levels.	[99]

**Table 2:** Chemical structures of bioactive compounds extracted from Ginkgo biloba sources and their effects on neurological disorders linked to COVID-19.

Compound	Sources	Activity/Mechanism	Ref.
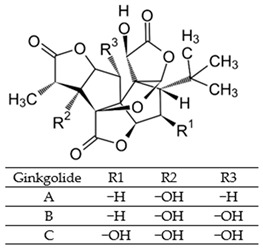	Leaves, root, and bark	Anti-inflammatory actions by decreasing the expression of TNF-α, IL-1β, and NF-kB Antioxidant effects by inhibiting free radicals Anxiolytic-like effects Coffer neuroprotection by regulating pathways related to neurodegeneration and inflammation Anti-thrombotic action in inhibiting platelet aggregation by MMP-9 Controlling cAMP, thereby inhibiting intracellular Ca2+ mobilization and decreasing TXA2 activity Inhibited activation factor of platelets and signaling pathways, such as NIK/IKK α/I-kβ/NF-κB Activation of the p42/p44 (ERK) MAPK pathways, effect on mRNA levels and the protein from HIF-1α	[120,121,122,123,124,125,126]
Bilobalide 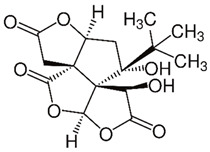	Leaves and bark	Decreased TNF-α, IL-1β, and IL-6 levels Neuroprotection by reducing neuroinflammation and preventing Aβ deposition in AD Antioxidant effects by decreasing ROS Modulation of Bax, c-myc, and p53 proteins Activated Nrf2 and CREB through the PI3K/Akt signaling pathway, thus inhibiting apoptotic damage of nerve cells	[119,127,128]
Isorhamnetin 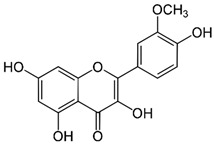	Leaves	Improved brain function and cognition Anti-inflammatory/antioxidant properties Decreased apoptosis and DNA fragmentation Cleavage of PARP, impact on the ERK pathway and the activation of p53 protein Upregulation of genes related to Bcl-2 Downregulation of the BH3 gene and genes related to Bax	[117,129,130,131]
Ginkgolic acid 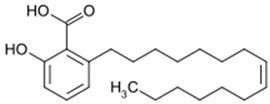	Leaves	Neuroprotection against Aβ-induced impairment of neurotransmitter release and synaptic plasticity Antiviral and antibacterial effects by suppressing the fusion of enveloped viruses, including SARS-CoV-2 Promotion of autophagy-dependent clearance of α-syn aggregates	[132,133]
Quercetin 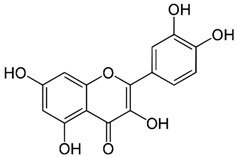	Leaves	Upregulated BDNF levels Inhibited degradation of serotonin by monoamine oxidases Impact on the transcription of TNF-α Activation of ERK and JNK Decreased lipid peroxidation in the plasma and phosphorylation of I-kβ Upregulation of HMOX-1 Free-radical elimination	[129,134,135,136]
Luteolin 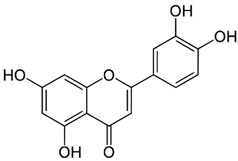	Leaves	Anti-inflammatory effect by suppressing TNF-α, IL-6, COX-2, and NF-kB expression Antioxidant effect by scavenging ROS Neuroprotective action by inhibiting Aβ deposition and augmenting neuroinflammation in the brain	[137,138]
Kaempferol 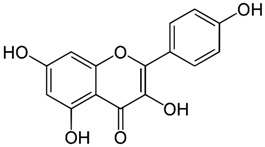	Leaves	Upregulation of GSH and suppression of oxidative and inflammatory damage to brain cells Inhibition of NF-kB, COX-2, and iNOS expression Protection against ischemia/reperfusion syndrome and myocardial injury Upregulation of BDNF, GCLC, and Bcl-2 levels Decrease in neurotoxicity induced via 3-NP, elevation of Bax, and upregulation of HMOX-1	[104,105,107]

**Table 3:** In vivo and in vitro evaluation of bioactive constituents of Ginkgo biloba and their neuroprotective role under neurodegenerative conditions.

Ginkgo biloba Constituents	Neurological Condition	Model	Outcome	Ref.
EGb 761	Age-associated mitochondrial dysfunction	SAMP8 mice, oral administration	Protection against mitochondrial dysfunction in platelets of young and old mice Reduction in ROS-induced apoptosis	[153,154]
Ginkgetin and bilobalide	PD	MPTP-induced mice, oral administration	Eliminated neuroinflammation via decreasing TNF-α levels Increased levels of BDNF in the substantia nigra pars compacta Decreased levels of intracellular ROS and maintained mitochondrial membrane potential Inhibited cell apoptosis via caspase-3 and Bcl2/Bax pathways Increased tyrosine hydroxylase expression in the substantia nigra and SOD activity in the striatum Chelated iron ions, downregulated L-ferritin, and upregulated transferrin receptor 1	[155,156]
Ginkgo biloba extract (EGb LI 1370)	AD	SH-SY5Y cells expressing amyloid precursor protein (APP)	Decreased oxidative stress Ameliorated oxidative phosphorylation and restored Aβ-induced deficits Improved oxygen consumption and upregulation of mitochondrial DNA	[157]
Ginkgo biloba supplements (GBS)	PD	Rotenone-induced Swiss mice, oral administration	Inhibited striatal dopaminergic neurodegeneration and α-synuclein immunoreactivity Suppressed executioner caspase-3 and upregulated Nrf2 pathway Decreased neurodegeneration of somata size and dendritic spine of striatal neurons	[158]
Ginkgo biloba dropping pill (GBDP)	PD	In vivo: 6-OHDA-induced zebrafish MPTP-induced male C57BL/6 mice, oral administration In vitro: MPP+-induced SH-SY5Y cells	Protection of dopaminergic neurons against 6-OHDA and MPTP-induced neurotoxicity mediated by the Akt/GSK3β signaling pathway Reduced cognitive impairments and neuronal damage	[152]
Ginkgo biloba tablets	Vascular cognitive impairment of non-dementia (VCIND)	Randomized clinical study of 80 patients with VCIND	Significant improvement in the Montreal Cognitive Assessment (MoCA) score Increased blood flow velocity of the anterior cerebral artery	[159]
EGb (Symfona^®^ forte 120 mg)	Mild cognitive impairment (MCI)	Randomized, double-blind, placebo exploratory study in 50–85-year-old patients with MCI and associated dual-task-related gait impairment	Increased dual-task-related performance in the intervention group EGb-associated numerical non-significant trends found after 6 months for dual-task-related gait velocity and stride time variability	[150]
EGb 761	Huntington’s disease	3-NP-induced rats, I.P. injection	Downregulation of striatal Bax Upregulation of striatal Bcl-xl expression level	[105,160]
Kaempferol and luteolin	AD	Transgenic drosophila expressing wild-type human Aβ42	Improved memory Reduced oxidative stress and acetylcholinesterase activity Inhibition of Aβ42 plaque formation after binding to Aβ42 Inhibition of AChE Increased GHS content	[137,161]
Bilobalide	Cerebral ischemia and reperfusion (I/R) injury	MCAO male Sprague–Dawley rats	Significantly decreased infarct volume, brain edema, MDA, nitric oxide, TNF-α, and IL-1β Increased SOD activity Downregulated p-JNK1/2 and p-p38 MAPK expression	[162]
Isorhamnetin	Ischemia-induced cerebral vascular degeneration	Human brain microvascular endothelial cells (HBMECs)	Reduced activation of the extrinsic apoptotic pathway by decreasing caspase-3 and caspase-8 Inhibition of FAS/FASL expression and suppressed NF-κB nuclear translocation	[163]
EGb 761	Ischemic brain injury	MCAO male Sprague–Dawley rats	Decreased parvalbumin expression	[164]
Ginkgolide B (GB)	Vascular dementia (VD), hypoxic injury	In vivo: BCCAO rats, intraperitoneal injection In vitro: Oxygen-glucose deprivation (OGD) in SH-SY5Y cells, primary hippocampal neurons subjected to chemical hypoxia (0.7 mM CoCl_2_)	Reduced TLR4/NF-κB-mediated neuroinflammation Regulated Ca2^+^ influx and homeostasis Decreased number of apoptotic cells in different areas of the hippocampus Improved antioxidant defense system (SOD, GSH, CAT) Decreased concentration of malondialdehyde (MDA) in the rat hippocampus	[165,166,167]

**Table 4:** Preparation, characterization, and evaluation of active constituents of Ginkgo-biloba-based nanoparticles for treatment of neurological damages linked to COVID-19.

Molecule	Nanocarrier	Technique	ζ-Potential, mV	Size, nm	PDI	DL%	EE%	Morphology	Pathology	Ref.
Ginkgolide B	GB–PPNPs	Antisolvent precipitation	−10.37 ± 0.56	77.58 ± 0.77	0.124 ± 0.018	19.43%	92.08	Spherical	PD	[198]
Quercetin	QNPs	Antisolvent precipitation	—	<1000	0.734	—	—	—	AD	[203]
Quercetin	QT–SPION conjugates	Co-precipitation technique	—	30–50	—	—	—	Spherical	AD	[204]
Ginkgo biloba extract	EGb niosomes	Freeze-drying and spray-drying methods	Noisome suspension, −0.1 ± 1.7 Freeze-drying, −11.6 ± 4.3 Spray-drying, −33.6 ± 1.6	141.3 ± 11.9 661.3 ± 78.6 680.2 ± 90.0	—	—	50.0 ± 1.9 50.1 ± 1.0 77.5 ± 1.0	Spherical and smooth surface	Improving oral bioavailability	[205]
Kaempferol	Kaempferol-loaded nanoparticles (KFP–NPs)	Quasi-emulsion methods	−28.5 to −7.5	201 ± 0.45	0.12 to 0.95	11.34 to 15.06	30.14 to 46.72	Solid sphere with a smooth surface	Hepatoprotective and antioxidant effects	[206]
Luteolin	Luteolin-loaded chitosomes (LUT–CHS)	Ethanol injection	37.4 ± 2.13	412.8 ± 3.28	0.378 ± 0.07	—	86.6 ± 2.05	Spherical vesicular system with a phospholipid bilayer membrane	Cognitive dysfunction in Alzheimer’s disease (AD)	[207]
Ginkgo biloba	EGb-loaded solid lipid nanoparticles (SLNs)	High-pressure homogenization	−12.6 to −28	104 to 621	< 0.5	—	79 to 89	Spherical, smooth, and rounded surface	Cytotoxicity and antibacterial activities	[196]
Isorhamnetin	Isorhamnetin-PLGA NPs	Double-emulsion solvent evaporation	—	255 to 342	—	—	—	—	—	[208]
Ginkgo biloba extract	Gb-extract -loaded chitosan nanoparticles (Gb–CsNPs)	Ionic gelation	29.3	104.4	0.09	40	97.4	Smooth and spherical morphology	Oxidative stress	[192]
	Silver nanoparticles (AgNPs)	Biogenic synthesis	−74.2 ± 2.45	5.46 to 19.40	—	—	—	Agglomerated spherical shapes	Antiviral activities against MERS-CoV and HCoV-229E	[202]
	Self-emulsifying drug delivery systems (SEEDS)	Self-emulsification	—	~100	—	—	—	—	Improving oral absorption	[209]
	EGb-loaded nanospheres	Nanoprecipitation	—	100 to 200	0.428 to 0.478	—	—	Oval or spherical shape with a smooth surface	In vitro release kinetics	[210]

**Table 5:** Nanospecific characteristics and physico-chemical properties of drug-loaded nanoparticles, which are considered relevant for the preclinical characterization of nanomedicines/nanotherapeutics formulated in aqueous media.

Nanospecific Characteristics	Test Method
Size/size distribution	DLS
Physical form/shape/morphology	TEM, cryo-TEM
Surface charge	Zeta potential, electrophoretic mobility (EPM)
Aggregation behaviour	DLS
Stability and uniformity	DLS, UV–VIS spectroscopy
Density/weight/volume fraction of nanomaterial dispersed in the medium	Ultracentrifugation, densitometry
Drug encapsulation	UV–VIS spectrometry, high-performance liquid chromatography (HPLC)
Presence of targeting moieties	Kinetic turbidity assays, Spectroscopic assays (UV–VIS, circular dichroism), surface plasmon resonance (SPR) binding assays
Toxicity	Cytotoxicity assessment using MTT and LDH assays
Biocompatibility	Immunological response, hemolytic properties
Structural and functional properties	TEM, SEM, small-angle X-ray scattering (SAXS), NTA, high-resolution transmission electron microscopy (HRTEM), atomic force microscopy (AFM), extended X-ray absorption fine structure (EXAFS), ferromagnetic resonance (FMR), DSC, differential centrifugal sedimentation (DCS), inductively coupled plasma atomic emission spectroscopy (ICP-MS), UV–VIS, matrix-assisted laser desorption/ionization (MALDI), nuclear magnetic resonance (NMR), superparamagnetic relaxometry, tunable resistive pulse sensing (TRPS)

## Data Availability

The authors confirm that the data supporting the findings of this study are available within the article.

## Abbreviations

3-NP—3-nitropropionic acid; 6-OHDA—6-hydroxydopamine; 8-OHG—8 hydroxyguanosine; ACE2—angiotensin-converting enzyme 2; AKT—protein kinase B; ALI—acute lung injury; ALS—amyotrophic lateral sclerosis; AMPA—α-amino-3-hydroxy-5-methyl-4-isoxazole propionic acid; ARE—antioxidant response element; AT1R—angiotensin II receptor type 1; Aβ—amyloid beta; BBB—blood–brain barrier; BCL2—B-cell lymphoma-2; B-CSF—blood–cerebrospinal fluid; BDNF—brain-derived neurotrophic factor; cAMP—cyclic adenosine monophosphate; CAT—catalase; CDK5—cyclin-dependent kinase 5; CNS—central nervous system; COX2—cyclooxygenase-2; CREB—cAMP-response-element-binding protein; CVO—circumventricular organ; DCN—dopaminergic neuron; ECT—electron transport chain; EGb—Ginkgo biloba extract; EGCG—epigallocatechin gallate; eNOS—endothelial nitric oxide synthase; ER—endoplasmic reticulum; ERK—extracellular-signal-regulated kinase; FOXO3—forkhead box O3; GA—ginkgolide A; GCLC—glutamate-cysteine ligase catalytic subunit; GCLM—glutamate cysteine ligase modifier; GPx—glutathione peroxidase; GSH—glutathione; GSK-3β—glycogen synthase kinase-3 beta; hBMVEC—human brain microvascular endothelial cell; HIF-1—hypoxia inducible factor 1; HMOX1—heme oxygenase 1; hNPC—human neural progenitor cell; HO-1—*heme oxygenase*; I-Kβ—Ikappa B kinase; IL-1β—interleukin 1 beta; JNK—c-Jun N-terminal kinase; LPS—lipopolysaccharide; LRRK2—leucine-rich repeat kinase 2; MAM—mitochondria-associated endoplasmic reticulum membrane; MAPK—mitogen-activated protein kinase; MAPT—microtubule-associated protein tau; MCAO—middle cerebral artery occlusion; MDA—malondialdehyde; MERS-CoV—Middle East respiratory syndrome coronavirus; MPO—myeloperoxidase; MRI—magnetic resonance imaging; MtK_ATP_—ATP-sensitive potassium channels of the inner mitochondrial membrane; mtROS—mitochondrial reactive oxygen species; mTOR—mechanistic target of rapamycin; MTPT—1-methyl-4-phenyl-1,2,3,6-tetrahydropyridine; MYD88—myeloid differentiation primary response 88; nAChR—nicotinic acetylcholine receptor; NADPH—nicotinamide adenine dinucleotide phosphate; NF-κB—nuclear factor kappa B; NLRP3—NOD-like receptor proteins 3; NMDA—N-methyl-D-aspartic acid; NOX—NADPH oxidase; NQO1—NAD(P)H quinone dehydrogenase 1; Nrf2—nuclear factor erythroid 2–related factor 2; PARP—poly (ADP-ribose) polymerase; PD—Parkinson’s disease; PGC-1α—peroxisome proliferator activated receptor gamma coactivator 1 alpha; *PI3K*—phosphoinositide 3-kinase; PINK1—PTEN-induced kinase 1; PKB—protein kinase B; PRDX3—peroxiredoxin 3; PSEN1—presenilin 1; PTEN—phosphatase and tensin homolog; RBD—receptor-binding domain; ROS—reactive oxygen species; SARS-CoV-2—severe acute respiratory syndrome coronavirus 2; SOD2—superoxide dismutase 2; STAT3—signal transducer and activator of transcription 3; TLR4—toll-like receptor 4; TNF-α—tumor necrosis factor alpha; TXA2—thromboxane A2; γGCS—gamma glutamylcysteine synthetase; ΔΨm—mitochondrial membrane potential
